# Innovative Approaches to Regenerate Enamel and Dentin

**DOI:** 10.1155/2012/856470

**Published:** 2012-05-14

**Authors:** Xanthippi Chatzistavrou, Silvana Papagerakis, Peter X. Ma, Petros Papagerakis

**Affiliations:** ^1^Department of Orthodontics and Pediatric Dentistry, School of Dentistry, University of Michigan, Ann Arbor, MI 48109, USA; ^2^Department of Otolaryngology, Head and Neck Surgery and Oncology, School of Medicine, University of Michigan, Ann Arbor, MI 48109, USA; ^3^Department of Biological and Materials Sciences, School of Dentistry, University of Michigan, Ann Arbor, MI 48109, USA; ^4^Center for Organogenesis and Center for Computational Medicine and Bioinformatics, School of Medicine, University of Michigan, Ann Arbor, MI 48109, USA

## Abstract

The process of tooth mineralization and the role of molecular control of cellular behavior during embryonic tooth development have attracted much attention the last few years. The knowledge gained from the research in these fields has improved the general understanding about the formation of dental tissues and the entire tooth and set the basis for teeth regeneration. Tissue engineering using scaffold and cell aggregate methods has been considered to produce bioengineered dental tissues, while dental stem/progenitor cells, which can differentiate into dental cell lineages, have been also introduced into the field of tooth mineralization and regeneration. Some of the main strategies for making enamel, dentin, and complex tooth-like structures are presented in this paper. However, there are still significant barriers that obstruct such strategies to move into the regular clinic practice, and these should be overcome in order to have the regenerative dentistry as the important mean that can treat the consequences of tooth-related diseases.

## 1. Introduction

Enamel is the outermost covering of vertebrate teeth and the hardest tissue in the vertebrate body. During tooth development, ectoderm-derived ameloblast cells create enamel by synthesizing a complex protein mixture into the extracellular space where the proteins self-assemble to form a matrix that patterns the hydroxyapatite [1] woven to form a tough, wear-resistant composite material [2]. The mature enamel composite contains almost no protein [3] and is a hard, crack-tolerant, and abrasion-resistant tissue [4]. During enamel biomineralization, the assembly of the protein matrix precedes mineral replacement. The dominant protein of mammalian enamel is amelogenin, a hydrophobic protein that self-assembles to form nanospheres that in turn influence the crystal habit and packing of the crystallites [5]. In contrast to the mesenchyme-controlled biomineralization of bone, which uses collagen and remodels both the organic and inorganic phases over a lifetime, enamel contains no collagen and does not remodel.

Mineralized dentin is synthesized by odontoblasts that line the centrally located dental pulp chamber and is deposited beneath the enamel and cementum [6]. Dentin, otherwise to the enamel, is soft flexible and able to absorb energy, and resists fracture. It is less mineralized than enamel, and it is a sort of sponge crossed by channels of one micron wide radically departing from the odontoblasts. These channels called “dentinal tubules,” are occupied by a part of the odontoblasts whose cytoplasm body underlies the dentin-dental pulp interface. Dentinal fluids are also present in the tubules. Dentin is formed by mineralization of the dentin matrix mainly composed of collagen type I and some specific noncollagenous matrix proteins. The deposition of the dentin occurs over the life of the teeth. Sometimes in the immature dentin appear globules which are fusing during the maturation of the tissue [7]. Odontoblasts can be formed from dental pulp stem cells following a differentiation process induced by required signals [8]. It is also known that, in response to stimulation with recombinant BMPs, dental pulp cells differentiate into dentin-forming odontoblasts [9]. However, it is still unknown what is the required ideal combination of signals and the minimum set of cells, to engineer all the cellular components of a fully functional dental pulp, while the allegation that dental pulp stem cells may have the potential to differentiate into most cells of the dental pulp has not yet been strongly demonstrated in vivo.

Operative dentistry has been using regenerative processes to treat dental disease. The use of calcium hydroxide to stimulate reparative dentin is an example of therapeutic strategy. Tissue engineering enhances dentistry to move forward in the application of regeneration as important principle for the treatment of dental disease. It is based on fundamental approaches that involve the identification of appropriate cells, the development of conductive biomaterials, and an understanding of the morphogenic signals required to induce cells to regenerate the lost tissue. Extended research has started to emerge in the field of enamel and other dental tissue regeneration applying material-cell-based strategies. It is expected that strategies involving the use of tissue engineering, nanotechnology, and stem cells to have an increasing participation in clinical dentistry over the next 5–20 years [10]. There are major issues to overcome before such strategies be introduced into the clinic and used regularly to treat dental diseases. However, there is evidence that suggest tissue engineering as the main approach in the future of operative dentistry, for the development of new dental structures.

## 2. Making Enamel

Odontoblasts are found in the dental pulp of erupted teeth. In their absence, undifferentiated dental pulp cells or dental pulp stem cells can be differentiated into odontoblasts and restore the capability of the dental pulp to synthesize reparative dentin. However, ameloblasts which specialize in making enamel are not present in teeth with complete crown development. Consequently, an endogenous regeneration of enamel is not feasible, while the development of synthetic enamel and/or in situ cell-based approaches are being achieved by using the principles of tissue regeneration and nanotechnology.

### 2.1. Restoration: Synthetic Enamel Fabrication

**Figure 1:**
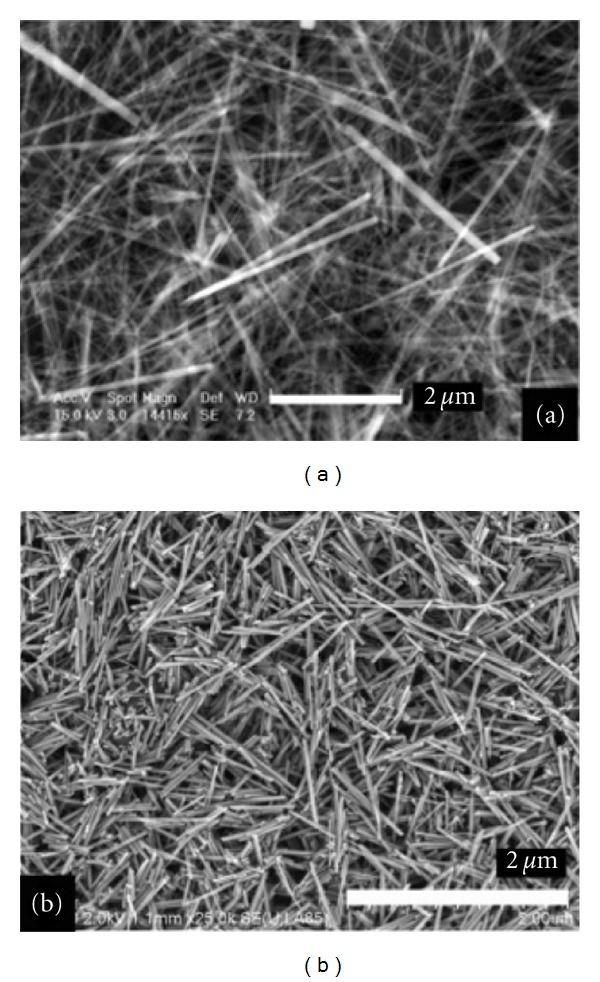
SEM images of (a) fluorapatite nanorods prepared by direct precipitation from solution and (b) enamel crystals isolated from the maturation stage of rat incisor enamel [8]. (Reproduced with permission from the American Chemical Society.)

Surfactants were used as reverse micelles or microemulsions to synthesize enamel, as they can mimic the biological action of enamel proteins [11]. The synthesized nanoscale structures may self-assemble into “one dimensional building blocks” leading to the development of hydroxyapatite nanorods similar to natural enamel crystals. The fabricated nanorods can potentially be applied as flowable restorative material for the restoration of lost enamel. Chen et al. [12] based on the biological processes involved in amelogenesis, combined with new approaches in nanotechnology, fabricated enamel prism-like structures consisted of fluorapatite nanorods (Figure 1(a)) precipitated directly from solution under controlled chemical conditions without the use of surfactants, proteins, or cells. The fabricated nanorods present similar characteristics to those of the natural enamel crystals isolated from rat incisor enamel, as it is confirmed from the scanning electron microscope (SEM) images in Figure 1(b).

Another enamel-based biomaterial having the added benefit of fluorapatite incorporated intrinsically into the composition was also observed. Particularly, amelogenin-driven apatite crystal growth, incorporating fluoride into the process, allowed the synthesis of elongated rod-like apatite crystals with dimensions similar to those observed in natural enamel [13]. Although the extended research for engineering advanced biomaterials, it is evidenced that none of the available material today can mimic all the physical, mechanical, and esthetic properties of enamel. This conclusion was an important parameter toward the establishment of cell-based strategies that could stimulate enamel regeneration.

### 2.2. Regeneration: Cell-Based Strategies

It has been suggested that extracellular matrix proteins such as fibronectin [14], laminin [15], and ameloblastin [16] not only function as a mechanical scaffold for cell attachment and survival but also provide a microenvironment for guiding cell growth and differentiating on. Considering this suggestion Huang et al. used an in vitro cell and organ culture system, to study the effect of artificial bioactive nanostructures on ameloblasts with the long-term goal of developing cell-based strategies for tooth regeneration. Particularly, a branched peptide amphiphile molecules containing the peptide motif Arg-Gly-Asp or “RGD” (abbreviated BRGD-PA), known to self-assemble in physiologic environments into nanofibers network, was used in order to mimic the extracellular matrix that surrounds the ameloblasts. Ameloblast-like cells (line LS8) and primary enamel organ epithelial (EOE) cells were cultured within BRGD-PA hydrogels and formed focal multilayered structures that accumulated minerals [17]. BRGD-PA was also injected into the enamel organ epithelia of mouse embryonic incisors. At the site of injection, it was observed EOE cell proliferation with differentiation into ameloblasts as evidenced by the expression of enamel-specific proteins [17]. Moreover, it was shown the nanofibers within the forming extracellular matrix, in contact with the EOE cells engaged in enamel formation and regeneration. Finally it was concluded that BRGD-PA nanofibers present with enamel proteins participate in integrin-mediated cell binding to the matrix with delivery of instructive signals for enamel formation [17].

## 3. Making Dentin

A crosstalk that involves signals of diffusible molecules from the epithelium induces odontoblasts to synthesize extracellular matrix proteins required for dentin formation [18]. There is a big research in the field of the different inducers of dentin mineralization. The demineralized dentin powder, likewise the demineralized bone powder, observed to have also the capability to induce mineralization when applied directly to areas of pulp exposure [19, 20]. Specific functions of dentin seem to contain bone morphogenetic protein (BMP) activity, which induces reparative dentin formation, leading to the potentially use of BMPs in dentin regeneration [16, 20, 21].

Moreover the use of recombinant human proteins combined with collagen-based matrixes was applied to induce dentin regeneration. It was observed the induction of reparative dentin at the sites of pulp exposure within a period of 2 to 4 months [22, 23]. The general mechanism of this process is based on the fact that reparative dentin is formed where the stimulating agents were placed in direct contact with the dental pulp. This consideration was strengthened as it was observed a proportional dependence of the area of the induced reparative dentin with the amount of the applied BMP-7, which could eventually allow the predetermination of dentin's amount [24]. However the induction of reparative dentin was not successful in the case of inflamed dental pulps, which was assigned to insufficient amount of active recombinant protein due to its relative short half-life and to the faster degradation rates of the protein in the presence of the inflamed pulp [25].

The capability to induce reparative dentin was also found to growth/differentiation factor 11 (Gdf11) with a direct delivery to pulp cells applying a gene transfer strategy [26]. Additionally, bone sialoprotein (BSP) was observed to stimulate the differentiation of dental pulp cells into cells that can secrete extracellular matrix which is further mineralized into reparative dentin, presenting different morphological characteristics compared to the respective induced by BMP proteins [27]. This observation enhances the consideration that one day based on the patient's needs it will be possible to have the capability to select the ideal type of biological inducer for the desired reparative dentin.

**Figure 2:**
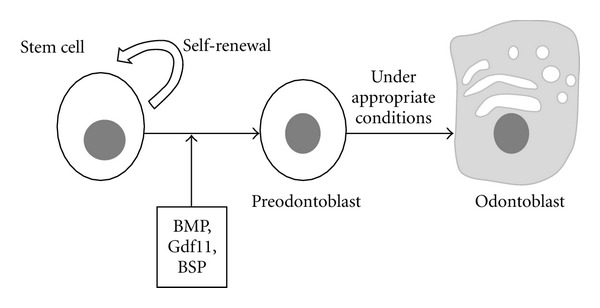
Differentiation of stem cell induced by appropriation signals such BMPs, Gdf11, or BSP into preodontoblast which can differentiate into odontoblast which can finally regenerate dentin.

In addition, the side population fraction of human dental pulp cells and the periodontal tissue stem cells derived from human-extracted teeth observed to partially regenerate dentin and periodontal tissue by cell transplantation into defects [28], suggesting that the transplantation of stem cells for partial tissue repair using autologous dental tissue stem/progenitor cells is possible when appropriate signals coexist, as it is schematically presented in Figure 2. These cells are thought to be already committed to dental cell lineages as they are able to form dental tissues without epithelial-mesenchymal interactions. In addition to specific cells and signaling molecules, the importance of scaffolds in guiding dentin regeneration has also been evaluated [29].

## 4. Current Research in Jointed Dentin-Enamel Regeneration

**Figure 3:**
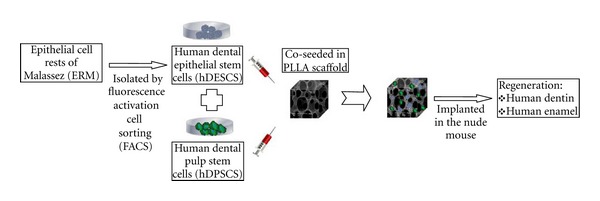
Layout of a cell-based strategy for the development of complex-like mineralized tissue by the co-seeding of hDESC and hDPSC.

Tissue engineering using scaffold and cell aggregate methods has been also suggested to produce bioengineered complex dentin-enamel regeneration from dissociated cells. Shinmura et al. [30] investigated the capability of epithelial cell rests of Malassez (ERM) to regenerate dental tissues by transplanting subcultured ERM seeded onto scaffolds into the omentum of athymic rats. Particularly, in combination with dental pulp cells at the crown formation stage, ERM was coseeded into collagen sponge scaffolds. After 8 weeks transplantation, enamel-dentin complex-like structures were recognized in the implants, as enamel-like tissue and the stellate reticulum-like structures were observed to some degree, while the tall columnar ameloblast-like cells were aligned with the surface of the enamel-like tissues. Similar results were observed in our lab with dental epithelial stem populations isolated by fluorescence activation cell sorting (FACS) using previously discovered epithelial stem cell markers [31] and subcultured under serum-free and xenon-free conditions. As it is illustrated in Figure 3, the collected human dental epithelial stem cells (hDESCs) can generate mineralized tissue in vivo when coseeded on PLLA scaffolds with human dental pulp stem cells (hDPSCs) and implanted subsequently in the nude mouse. After 10 weeks postimplantation mineralization is seen in the implants. Furthermore, complex dental tissues regeneration was investigated with different types of reassociations between epithelial and mesenchymal tissues and/or cells from mouse embryos which were cultured in vitro before in vivo implantation. In vitro the reassociated tissues developed and resulted in jointed dental structures that exhibited normal epithelial histogenesis and allowed the functional differentiation of odontoblasts and ameloblasts. After implantation, the reassociations formed roots and periodontal ligament, the latter connected to developing bone [32].

## 5. Conclusions: Future Trends

Regeneration of tooth parts is a complex attempt [33]. The treatment of tooth with inflamed pulp is considered as a main difficult challenge. A potential solution could be the application of appropriate advanced biological systems with therapeutic agents able to control the inflammatory response while inducing mineralization. An additional important challenge is the development of suitable carriers which can house all the necessary factors for the treatment and regeneration of lost/diseased tooth parts, while they should present biocompatibility, physicochemical, and mechanical properties compatible to their application in restorative dentistry. These new fabricated carriers should be able to create well-sealed restorations, preventing microleakages and subsequent contamination of the exposure pulp before the mineralization. The use of composites of synthetic or natural 3D scaffolds with bioactive antibacterial materials seeded with specific dental tissue stem cells could be a potential innovated system fulfilling all these significant requirements. Consequently, extended interdisciplinary research and effective collaboration between basic scientists and clinicians could potentially lead this field to the final goal of regeneration tooth parts or eventually the entire tooth.
